# Population genetic analysis of 12 X-chromosomal STRs in a Swiss sample

**DOI:** 10.1007/s00414-021-02684-y

**Published:** 2021-08-22

**Authors:** Michel Bottinelli, Alexandre Gouy, Silvia Utz, Martin Zieger

**Affiliations:** 1 Laboratorio di Diagnostica Molecolare, Via G. Petrini 2, 6900 Lugano, Switzerland; 2 Gouy Data Consulting, Sentier de Renges 4A, 1026 Denges, Switzerland; 3 Institute of Forensic Medicine, Forensic Molecular Biology Department, University of Bern, Sulgenauweg 40, 3007 Bern, Switzerland

**Keywords:** Switzerland, X-STR, Kinship, Population data, Forensics

## Abstract

X-chromosomal STRs are a powerful tool to assess a broad variety of complex kinship scenarios. We introduce herewith the first Swiss X-STR dataset based on 1198 individuals (592 female, 606 male), characterized with the Qiagen Investigator® Argus X-12 QS multiplex kit. Anomalous allele patterns, allele and haplotype frequencies, and forensic and population genetic parameters are presented. We detected linkage disequilibrium within three out of the four designated linkage groups and no apparent intra-national population substructure. We compared the dataset to a global panel of X-STR datasets and it fits well in the European context, as expected.

**Supplementary information:**

The online version contains supplementary material available at 10.1007/s00414-021-02684-y.

X-chromosomal STRs can be very helpful for the assessment of complex kinship scenarios. However, after 20 years of research and X-STR usage in forensic genetics, there is still a continuous need for high-quality population data [1]. With the present publication, we contribute the first-ever X-STR dataset for Switzerland.

We analyzed a sample of 1198 Swiss individuals (592 females, 606 males) with Qiagen Investigator® Argus X-12 QS multiplex kit in an ISO17025 accredited laboratory framework. DNA extractions were prepared as described in Zieger and Utz [2]. Multiplex PCR was performed in a reduced reaction volume of 12.5 μL. Capillary electrophoresis was conducted on a 3500xl genetic analyzer (ThermoFisher, USA) and data interpretation was carried out with Genemapper® ID-X, v1.4 (Thermo Fisher, US). Genotypes were exported from Genemapper and the export file was manually controlled twice by comparison with the electropherograms for QC. Details on the investigated population can be found in Zieger and Utz [2]. Allele frequencies and forensic parameters were calculated with StatsX [3]. Test for Hardy–Weinberg equilibrium (HWE) based on female samples with 100,000 permutations and calculation of pairwise *F*_ST_ among potential subpopulations were done with STRAF [4]. We checked for linkage disequilibrium with the genepop R package [5, 6] by performing an exact test with 50,000 iterations and 1000 batches. All statistics were calculated excluding eight genotypes with triallelic patterns.

Allele frequencies are listed in Table S1, haplotype frequencies in Table S2, and statistical parameters can be found in Table S3. Despite the low *p*-value of 0.006 for DXS7423, none of the 12 loci significantly deviates from HWE after correcting for multiple testing of 12 loci, based on a Bonferroni-adjusted threshold at 5% significance level *p* = 0.0008. Haplotype frequencies are available online as FamLinkX [7] input file (Table S4). In order to use the data for calculations with FamLinkX, it is possible to open the.sav file as a project in FamLinkX.

We could detect significant linkage disequilibrium within three out of four linkage groups. In total, six out of 12 expected allele combinations displayed significant linkage disequilibrium, after applying a Bonferroni correction with a significance threshold of 0.0008. Note that the Bonferroni correction assumes independence between tests and is overly conservative in the presence of linkage, so it is worthwhile to mention that all marginally significant tests (*p* < 0.01) correspond to combinations within and not between linkage groups (Table S5), supporting the consideration of loci within these four linkage groups as haplotypes for forensic calculations.

We discovered variant alleles in about 3% (*n* = 37) of all analyzed samples. All variants are listed in Table S6. Most of them have been described previously [8–17]. However, we list 12 alleles in Table S6 for which we could not find a reference to date. Half of them were in DXS10146, so a better allele coverage by the kit manufacturer would be desirable for this marker. In addition to frequent off-ladder alleles, a couple of multi-allelic patterns were discovered. Most of them (6 out of 9) are in DXS10079, a locus for which duplications can be observed frequently [9, 17]. Contrary to off-ladder alleles and allele duplications, obvious allele dropouts occurred scarcely, with just one partial dropout in DXS10101 and a potential dropout in DXS10146, inferred from the constantly reduced height of the remaining peak in a female sample. Dropouts have been reported previously for both of those markers [18].

We checked for potential intra-national differentiation by calculating pairwise *F*_ST_ values between subgroups defined geographically (for details, see Zieger and Utz [2]) based on female samples. Even though subsamples were relatively small (50 to 130 individuals), *F*_ST_ values are generally very low (not exceeding 0.004) and uniformly distributed, suggesting no significant degree of population stratification for this marker set (Table S7).

The allele frequencies of the complete Swiss dataset were compared to 36 other worldwide populations [8] using multidimensional scaling (MDS) based on Nei’s genetic distance [19]. The Swiss dataset clusters very well with other European datasets, as expected (Figure S8).

## Supplementary information

Below is the link to the electronic supplementary material.

Supplementary file1 (XLSX 26 KB)

Supplementary file2 (XLSX 29 KB)

Supplementary file3 (XLSX 14 KB)

Supplementary file4 (SAV 23 KB)

Supplementary file5 (DOCX 267 KB)

Supplementary file6 (DOCX 13 KB)

Supplementary file7 (DOCX 13 KB)

Supplementary file8 (DOCX 176 KB)

## References

[1] Gomes I, Pinto N, Antão-Sousa S, Gomes V, Gusmão L, Amorim A (2020). Twenty years later: a comprehensive review of the X chromosome use in forensic genetics. Front Genet.

[2] Zieger M, Utz S (2019). A “forensic biobank” to establish comprehensive genetic frequency data for Switzerland. Forensic Sci Int Genet.

[3] Lang Y, Guo F, Niu Q (2019). StatsX v2.0: the interactive graphical software for population statistics on X-STR. Int J Legal Med.

[4] Gouy A, Zieger M (2017). STRAF-A convenient online tool for STR data evaluation in forensic genetics. Forensic Sci Int Genet.

[5] Raymond M, Rousset F (1995). Genepop (version-1.2) - population-genetics software for exact tests and ecumenicism. J Heredity.

[6] Rousset F (2008). genepop’007: a complete re-implementation of the genepop software for Windows and Linux. Mol Ecol Resour.

[7] Kling D, Dell'Amico B, Tillmar AO (2015). FamLinkX - implementation of a general model for likelihood computations for X-chromosomal marker data. Forensic Sci Int Genet.

[8] Guo F (2017). Population genetic data for 12 X-STR loci in the Northern Han Chinese and StatsX package as tools for population statistics on X-STR. Forensic Sci Int Genet.

[9] Almarri MA, Lootah RA (2018). Allelic and haplotype diversity of 12 X-STRs in the United Arab Emirates. Forensic Sci Int Genet.

[10] Gomes I, Pereira PJP, Harms S, Oliveira AM, Schneider PM, Brehm A (2017). Genetic characterization of Guinea-Bissau using a 12 X-chromosomal STR system: inferences from a multiethnic population. Forensic Sci Int Genet.

[11] Becker D, Rodig H, Augustin C, Edelmann J, Götz F, Hering S, Szibor R, Brabetz W (2008). Population genetic evaluation of eight X-chromosomal short tandem repeat loci using Mentype Argus X-8 PCR amplification kit. Forensic Sci Int Genet.

[12] Ferragut JF, Bassitta M, Torrens V, Albeza V, Acreche N, Castro JA, Ramon C, Picornell A (2019). Analysis of 21 X-chromosome polymorphisms in urban and rural populations in Salta province (north-western Argentina). Int J Legal Med.

[13] Edelmann J, Lutz-Bonengel S, Naue J, Hering S (2012). X-chromosomal haplotype frequencies of four linkage groups using the Investigator Argus X-12 Kit. Forensic Sci Int Genet.

[14] Bini C, Riccardi LN, Ceccardi S, Carano F, Sarno S, Luiselli D, Pelotti S (2015). Expanding X-chromosomal forensic haplotype frequencies database: Italian population data of four linkage groups. Forensic Sci Int Genet.

[15] Salvador JM, Apaga DLT, Delfin FC, Calacal GC, Dennis SE, De Ungria MCA (2018). Filipino DNA variation at 12 X-chromosome short tandem repeat markers. Forensic Sci Int Genet.

[16] Rębała K, Kotova SA, Rybakova VI, Zabauskaya TV, Shyla AA, Spivak AA, Tsybovsky IS, Szczerkowska Z (2015). Variation of X-chromosomal microsatellites in Belarus within the context of their genetic diversity in Europe. Forensic Sci Int Genet.

[17] Mršić G, Ozretić P, Crnjac J, Merkaš S, Sukser V, Račić I, Rožić S, Barbarić L, Popović M, Korolija M (2018). Expanded Croatian 12 X-STR loci database with an overview of anomalous profiles. Forensic Sci Int Genet.

[18] Elakkary S, Hoffmeister-Ullerich S, Schulze C, Seif E, Sheta A, Hering S, Edelmann J, Augustin C (2014). Genetic polymorphisms of twelve X-STRs of the investigator Argus X-12 kit and additional six X-STR centromere region loci in an Egyptian population sample. Forensic Sci Int Genet.

[19] Nei M (1972). Genetic distance between populations. Am Nat.

