# Supplementary material for: Population genetic analysis of 12 X-chromosomal STRs in a Swiss sample
Source: Int J Legal Med. 2021 Aug 22;136(2):561–3. doi: 10.1007/s00414-021-02684-y (PMC8847170; doi:10.1007/s00414-021-02684-y)
Supplement: Supplementary file 5 — Supplementary file5 (DOCX 267 KB) [file 414_2021_2684_MOESM5_ESM.docx]

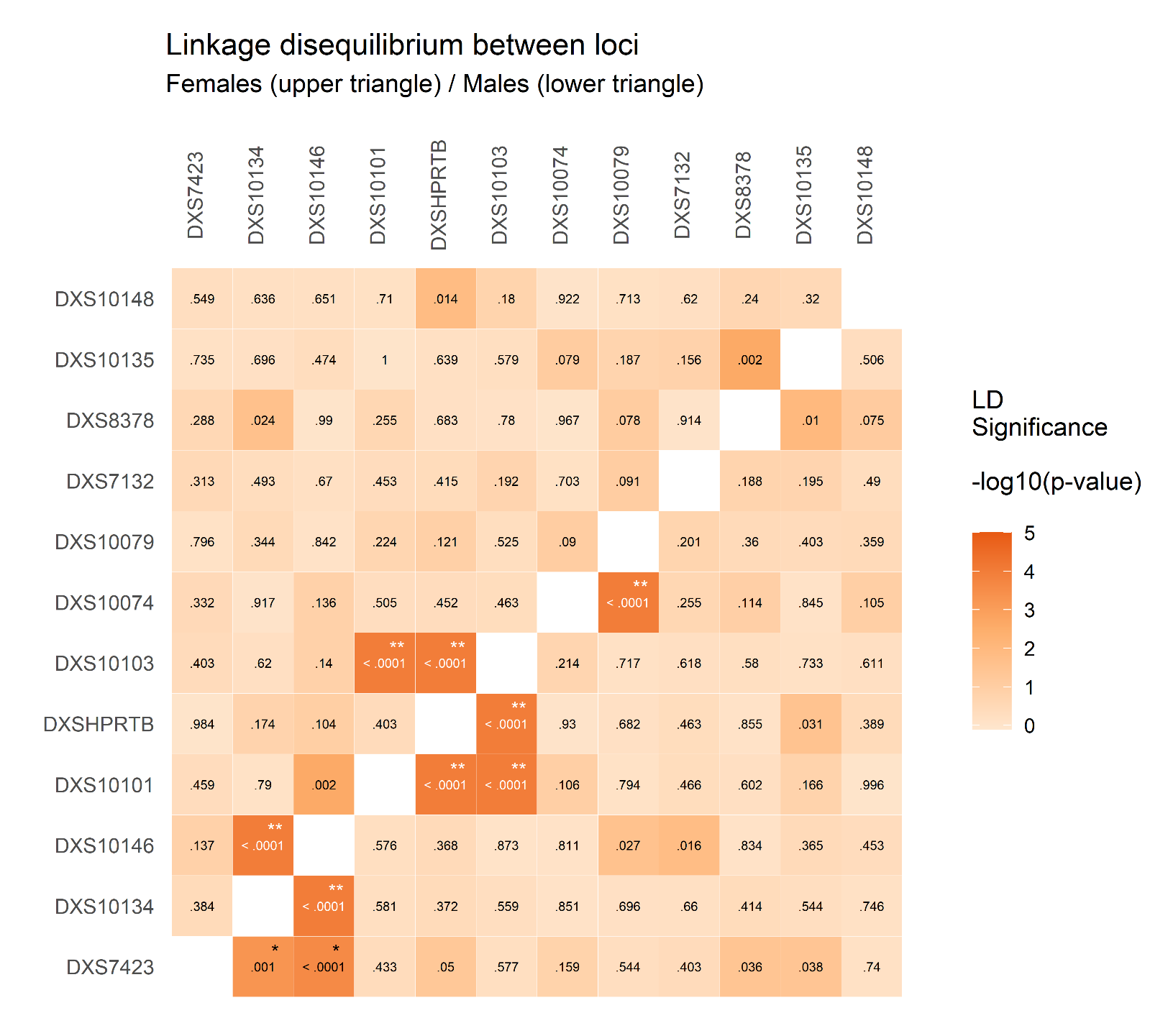


Table S5: Pairwise linkage disequilibrium test *p*-values, calculated separately for male (upper triangle) and female (lower triangle) samples. The p-values are rounded to the third decimal place and the color gradient is proportional to their significance (the darker, the more significant). Stars correspond to statistical significance after Bonferroni correction with 5 % (*) and 1 % (**) significance thresholds. Loci are ordered according to their position on the X chromosome.
