# Supplementary material for: Population genetic analysis of 12 X-chromosomal STRs in a Swiss sample
Source: Int J Legal Med. 2021 Aug 22;136(2):561–3. doi: 10.1007/s00414-021-02684-y (PMC8847170; doi:10.1007/s00414-021-02684-y)
Supplement: Supplementary file 6 — Supplementary file6 (DOCX 13 KB) [file 414_2021_2684_MOESM6_ESM.docx]

|  | **Off-ladder alleles** | **Multiple alleles** | **Allele dropout** |
| --- | --- | --- | --- |
| **DXS8378** | 7 | - | - |
| **DXS10101** | **23.3**  **30.1**  35.2 (2x) | - | M: partial dropout of allele 28 |
| **DXS10134** | **33.3**  36.1  45.3  **46.2** | - | - |
| **DXS10135** | **20.2**  **33.1** | - | - |
| **DXS10146** | **27.1**  **27.2** (4x)  29.3  36.2  38 (2x)  **38.3**  39.2  48.2 (2x)  **49** (3x)  **49.2**  **50.2** | F: 25/31/38  F: (23.2)/30/44.2* | F: potential dropout 35.2/- |
| **DXS10079** | 13  26 | F: 17/19/21  F: 20/21/22  M: 20/21  M: 20/22  M: 21/22  M: 21/23 | - |
| **DXS10148** | 24.2  25.2 (3x)  32.1  40.1 | F: 23.1/24.1/25.1* | - |

Table S6: Variant alleles. New off-ladder alleles are written in bold letters. F = female sample; M = Male sample; *unbalanced multi-allelic pattern (small additional allele in brackets).
