# Supplementary material for: Population genetic analysis of 12 X-chromosomal STRs in a Swiss sample
Source: Int J Legal Med. 2021 Aug 22;136(2):561–3. doi: 10.1007/s00414-021-02684-y (PMC8847170; doi:10.1007/s00414-021-02684-y)
Supplement: Supplementary file 7 — Supplementary file7 (DOCX 13 KB) [file 414_2021_2684_MOESM7_ESM.docx]

|  | NW | CS | BE | TI | WS |
| --- | --- | --- | --- | --- | --- |
| SG | 0.0038 | 0.0033 | 0.0026 | 0.0040 | 0.0029 |
| WS | 0.0023 | 0.0033 | 0.0023 | 0.0034 |  |
| TI | 0.0040 | 0.0039 | 0.0025 |  |  |
| BE | 0.0024 | 0.0031 |  |  |  |
| CS | 0.0036 |  |  |  |  |

Table S7: Pairwise F_ST_ values (female samples only). SG – St. Gallen (n=97); NW – Northwestern Switzerland (n=108); CS – Central Switzerland (n=80); BE – Bern (n=130); TI – Ticino (n=50); WS – Western Switzerland (n=125).
