# Supplementary material for: Population genetic analysis of 12 X-chromosomal STRs in a Swiss sample
Source: Int J Legal Med. 2021 Aug 22;136(2):561–3. doi: 10.1007/s00414-021-02684-y (PMC8847170; doi:10.1007/s00414-021-02684-y)
Supplement: Supplementary file 8 — Supplementary file8 (DOCX 176 KB) [file 414_2021_2684_MOESM8_ESM.docx]

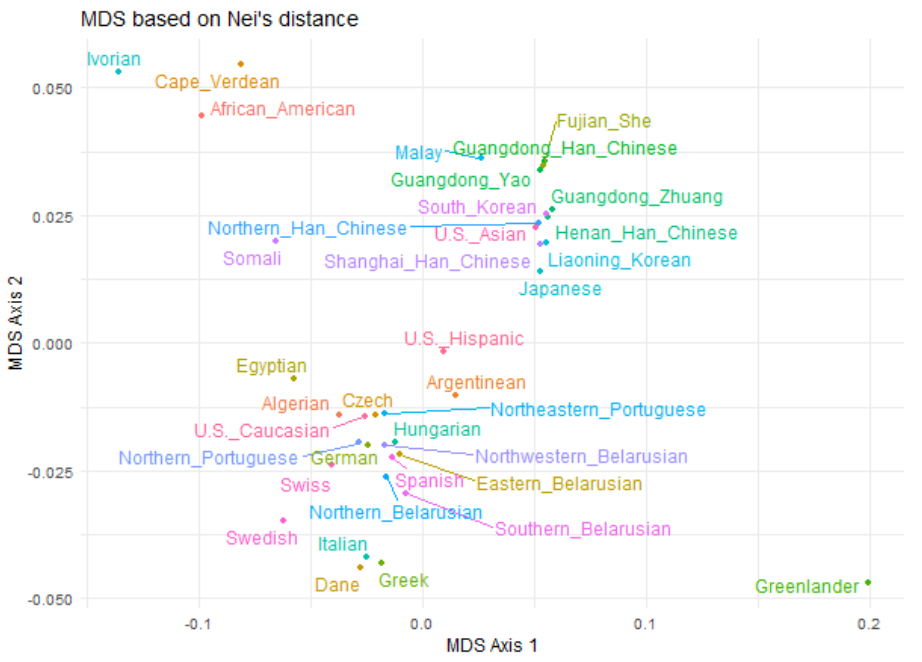


Figure S8: Multidimensional scaling (MDS) on the pairwise distance between populations, estimated using Nei’s genetic distance. Allele frequencies have been estimated in the full dataset (male and female samples from the Swiss population) and compared to all the other populations from F. Guo, FSI: Genetics 26 (2017) e1-e8.
